# Network-Based Bioinformatics Reveal Microenvironment-Driven Cell-to-Cell Communication in the Progression of Multiple Myeloma

**DOI:** 10.3390/ijms27114986

**Published:** 2026-05-30

**Authors:** Eleni Nicolaidou, Grigoris Georgiou, Anastasis Oulas, George M. Spyrou

**Affiliations:** Bioinformatics Department, The Cyprus Institute of Neurology and Genetics, 6 Iroon Avenue, 2371, Ayios Dometios, P.O. Box 23462, 1683 Nicosia, Cyprus; elenin@cing.ac.cy (E.N.); grigorisg@cing.ac.cy (G.G.); anastasioso@cing.ac.cy (A.O.)

**Keywords:** single-cell RNA sequencing, cell-to-cell communication, ligand–receptor pairs, network-based bioinformatics, tumour microenvironment, rewired cellular communication, downstream target genes, Multiple Myeloma

## Abstract

Single-cell RNA sequencing (scRNAseq) captures unique profiles of individual cells and uncovers cell-to-cell communication (CCC) through ligand–receptor (LR) interactions. Moreover, it reveals signalling mechanisms underlying cellular heterogeneity and complexity in downstream responses in healthy and disease states. In this work, we developed a composite computational pipeline to track CCC patterns in the tumour microenvironment (TME) during Multiple Myeloma (MM) progression as a case study. Three publicly available scRNAseq datasets were analysed using basic single-cell analytics and stage-specific CCC networks were reconstructed with CellChat, in a microenvironment-specific approach. Basic network analytics (CytoHubba) were performed to identify key cell nodes based on network topology metrics; differential network rewiring (DyNet) was performed to calculate rewired nodes. Follow-up analyses were conducted with NicheNet to investigate downstream responses and target genes influenced by CCC. Our network analyses highlighted dendritic cells (DCs), plasmacytoid DCs (pDCs), hematopoietic stem cells (HSCs), red pulp macrophages (RPMs), natural killer (NK) cells, and T and B cells as important cell nodes. Moreover, in neutrophils, the HLA-DRA–JUN–FOS was shown to play a key role in the progression of monoclonal gammopathies of uncertain significance (MGUS) to active MM by supporting cancer hallmarks and MM pathophysiology. To conclude, our work suggests an explanatory–computational pipeline that incorporates well-known frameworks in a hypothesis-driven scope, which leads to results relevant to the pathophysiology of MM.

## 1. Introduction

**Figure 1:**
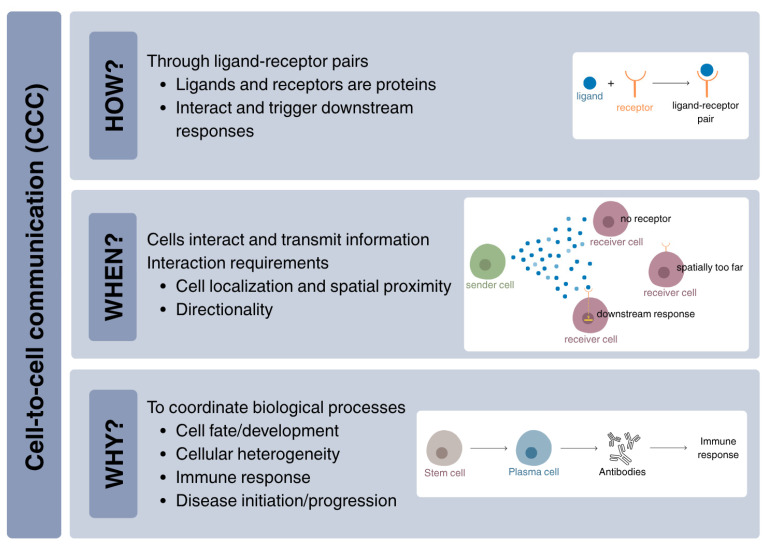
Overview of cell-to-cell communication mediated by ligand–receptor interactions. Cell-to-cell communication occurs through the binding of ligands to their cognate receptors, initiating downstream signalling cascades in recipient cells (HOW). Effective communication depends on spatial proximity between sender and receiver cells, appropriate cellular localization, and directionality of signalling (WHEN). These interactions regulate essential biological processes, including cell fate determination, maintenance of cellular heterogeneity, immune responses, and disease initiation and progression (WHY).

Cells coexist and collaborate in cell communities where they interact and communicate to contribute to the organism’s fine-tuning, a phenomenon known as cell-to-cell communication (CCC). CCC is mediated by interactions between ligand–receptor (LR) pairs, along with the involvement of co-factors, hormones, ions, neurotransmitters and cytokines [1]. For a cell-to-cell interaction to occur, a sender cell produces a ligand that diffuses towards its cognate receptor on the surface of the receiver cell. Then, a downstream response (e.g., activation of a signalling cascade) is triggered and further regulates downstream processes, such as the expression of a downstream target gene [2]. Interestingly, cellular proximity, affinity, directionality, and sufficient expression of the ligand and the receptor are some of the factors that influence the interactions between cells [3] (Figure 1).

The analysis of single-cell RNA sequencing (scRNAseq) data, although it provides an indirect measure of protein interactions, enables the understanding of cellular heterogeneity and complexity, tissue composition, cellular communication, and gene expression profiling at the resolution of individual cells [4]. Moreover, network biology is a field pivotal for understanding vital biological processes and functions at a systems level in both health and disease [5,6]. Currently, there is a wide abundance of publicly available toolkits and frameworks for the analysis and understanding of scRNAseq data in terms of CCC (e.g., CellChat, NicheNet) [2,7,8].

Multiple Myeloma (MM) is a plasma cell disorder accounting for 160,000 new cases globally and 1.1% of all cancer deaths [9]. Therefore, we aim to elucidate the intercellular relationships in MM in comparison to healthy control samples. MM is a hematologic malignancy that primarily affects the bone marrow and is characterized by cancerous myeloma plasma cells that produce abnormal immunoglobulins [10]. MM has three progressive stages: monoclonal gammopathies of uncertain significance (MGUS), Smouldering MM (SMM), and active MM.

Given that MM is initially characterised by the MGUS stage, which is a premalignant phase, some patients progress to SMM and finally to the disease stage, active MM. However, a small subset of MGUS individuals progress directly to MM without transitioning through the intermediate SMM stage [11]. Since we are interested in the progression of the disease, distinct analyses were performed in each dataset for the following progressing pairwise comparisons of interest: MGUS-MM and SMM-MM (when SMM was present).

Among our goals is to identify downstream target genes that are influenced by disease progression and may function as potential MM biomarkers. By incorporating scRNAseq data and analytics, along with several cell-to-cell inference frameworks, in this work, we aim to advance the understanding of LR pair-mediated interactions predicted by CellChat and ligand-to-target gene signalling paths inferred by NicheNet.

ScRNAseq studies on MM have characterised the bone marrow tumour microenvironment (TME) and identified key changes in immune and stromal cell populations during disease progression [12]. However, these studies often focus on individual datasets or isolated analyses, providing limited insight into how intercellular communication rewires across disease progression. Most current CCC analyses rely on individual computational tools such as CellChat (version 2.1), NicheNet (version 2.2.0), CellPhoneDB (version 5.0.0), and ICELLNET (version 1.3.0) [2,7,13,14]. These methods are typically applied as standalone frameworks with distinct assumptions and inference strategies. As a result, findings can vary depending on the tool used, with limited adoption of integrated multi-method approaches. To address this, we propose a composite computational framework that systematically incorporates multiple well-established CCC tools within a unified analytical workflow. This integrative strategy is inspired by emerging efforts such as LIANA and the work of Liu et al., which highlights the value of integrating methods to enhance robustness and interpretability, without highlighting a gold standard approach for CCC [15,16]. To our knowledge, such a multi-tool integration has not been systematically applied in MM, thereby providing a novel application of CCC analysis in this disease context.

We introduce a sequential computational pipeline that inputs already processed Seurat objects and studies cell-to-cell interactions, network topology, and rewiring and ligand-TF-target gene modelling. By applying this framework systematically across multiple independent datasets, our approach uncovers stage-specific rewiring of intercellular interactions and downstream transcriptional programs, revealing patterns that would not be apparent from individual tools or single-dataset analyses alone. This approach enables the identification of candidate biomarkers and provides a deeper understanding and insights into the disease’s evolution from a more systemic perspective, especially at the level of cellular interactions.

## 2. Results

### 2.1. Mapping CCC Dynamics Across MM Progression

CCC is a driving force that regulates several biological functions in multicellular organisms; therefore, we aimed to investigate how communication patterns change as MM progresses. Both unique and common cell types in the datasets are shown in Supplementary Figure S1. Six cell types were common across the three datasets: hematopoietic stem cells (HSCs), neutrophils, natural killer (NK) cells, plasmacytoid dendritic cells (pDCs), red pulp macrophages (RPMs), and T helper cells. Corresponding information is shown in Supplementary Table S1.

**Figure 2:**
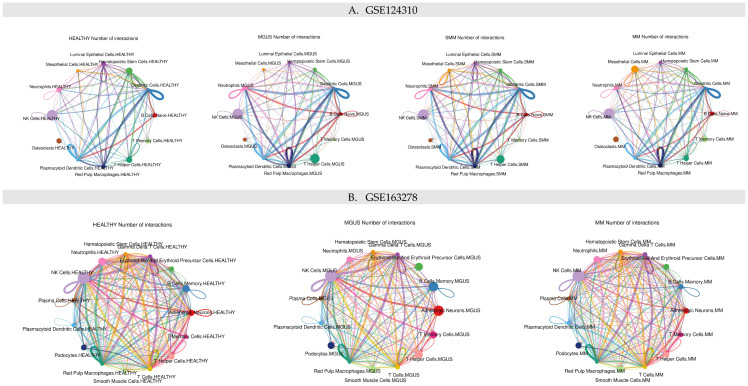

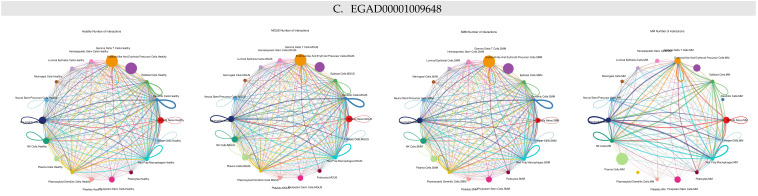
Cell-to-cell communication networks across disease states and datasets. (**A**–**C**) CCC networks inferred from ligand–receptor interactions in healthy, MGUS, SMM (when available), and MM samples across independent datasets. Nodes represent cell types, while directed edges indicate signalling from sender to receiver cells, with edge thickness reflecting interaction strength.

Weighted and directed CCC networks are shown in Figure 2. In the CCC networks, nodes represent cell types and their size is proportional to the cell population. The edges denote the number of LR interactions mediating the corresponding communication and are coloured according to the sender population cell node. The thickness of the edge is proportional to the number of LR pairs that mediate each interaction. Non-normalised and normalised cell proportions are summarised in Supplementary Tables S2 and S3.

### 2.2. DCs, pDCS, HSCs, RPMs, NK Cells, B Cells, and T Cells Were Recognized as Prominent Nodes Through Network Topology Analytics with CytoHubba

**Figure 3:**
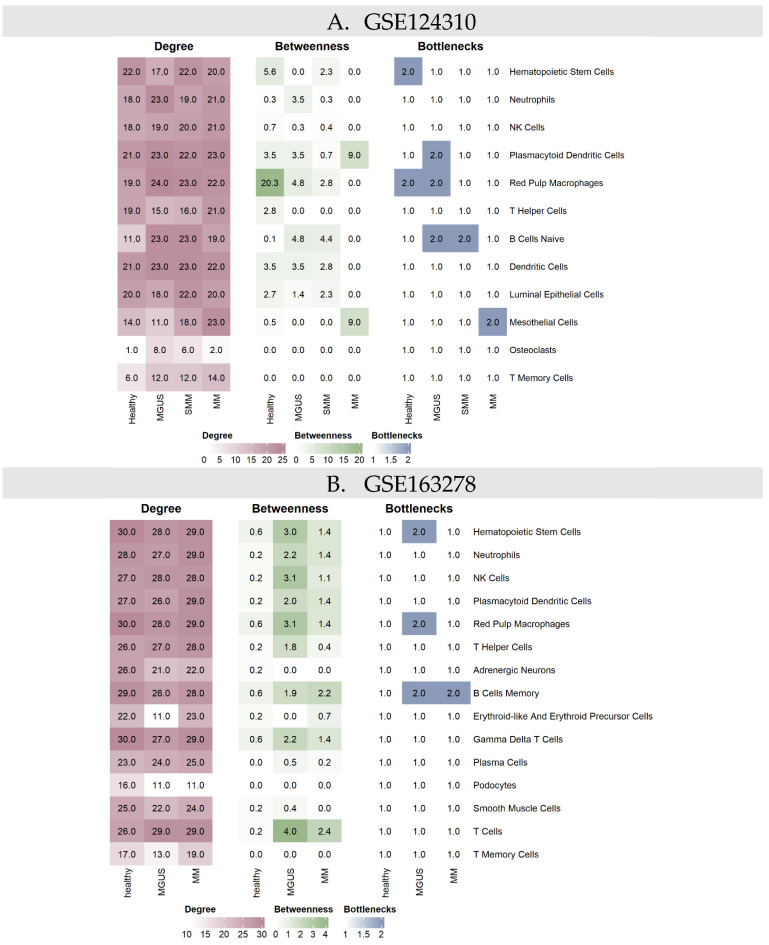

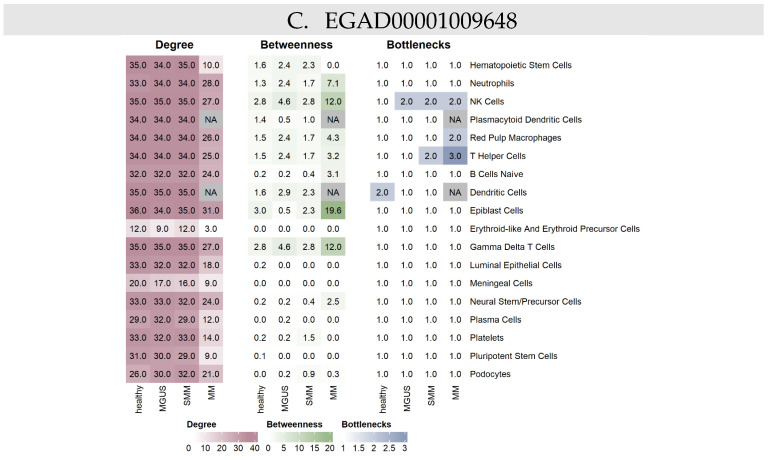
Network centrality metrics across datasets (**A**–**C**). Heatmaps showing degree, betweenness centrality, and bottleneck scores for each cell type across healthy, MGUS, SMM (when available), and MM conditions in independent datasets. Rows correspond to cell types and columns to disease states, with colour intensity indicating relative metric values. These analyses identify key cell populations with prominent roles in cell-to-cell communication networks. NAs indicate missing values, since these cell types have lost all their connections.

In the GSE124310 dataset, both naive B cells and T memory cells exhibited relatively low degree centrality in healthy networks, indicating limited connectivity at baseline (Figure 3A). However, their degree centrality increased across MGUS, SMM, and MM, suggesting that these immune populations become increasingly integrated into the global CCC architecture as disease advances. Overall, this trend indicates an expansion of network connectivity with progression, with a greater number of inferred interactions emerging in later disease stages. Moreover, RPMs in healthy networks exhibited a markedly elevated betweenness centrality score (20.3), indicating that RPM-associated interactions lie on a substantial fraction of shortest paths and suggesting that they act as key intermediates linking multiple cellular compartments. In the same dataset, the MGUS network showed enrichment of several bottleneck cell types, including pDCs, RPMs, and naïve B cells, highlighting these populations as critical connectors whose removal would be expected to disrupt network connectivity. In SMM networks, naïve B cells remained prominent bottlenecks, suggesting persistence of a structurally important B-cell-associated communication axis at this intermediate disease stage.

In the GSE163278 dataset, RPMs, NK cells, and T cells showed increased betweenness centrality in the MGUS network, indicating that these populations act as key intermediates through which a larger fraction of communication paths is routed at this stage (Figure 3B). Additionally, HSCs, RPMs, and memory B cells were identified as bottlenecks in MGUS, suggesting that they are important connectors that support overall network integrity. Notably, memory B cells remained prominent bottlenecks in the MM network, indicating that B-cell-associated communication continues to occupy a structurally important position in advanced disease.

In the EGAD00001009648 dataset, NK cells and γδ T cells exhibited increased betweenness centrality in the MM networks, suggesting that these populations occupy intermediate positions through which a substantial fraction of signalling paths are routed in the later disease stage (Figure 3C). Bottleneck analysis further indicated stage-specific shifts in critical connector populations: DCs were highlighted as bottlenecks in the healthy networks, whereas NK cells emerged as bottlenecks in MGUS. In SMM, both NK cells, T helper cells and RPMs indicate increasingly immune-centred and macrophage-linked communication routes with disease progression.

### 2.3. DCs, pDCs, Naïve B Cells, and T Memory Cells Show Extensive Network Rewiring During MM Progression

**Figure 4:**
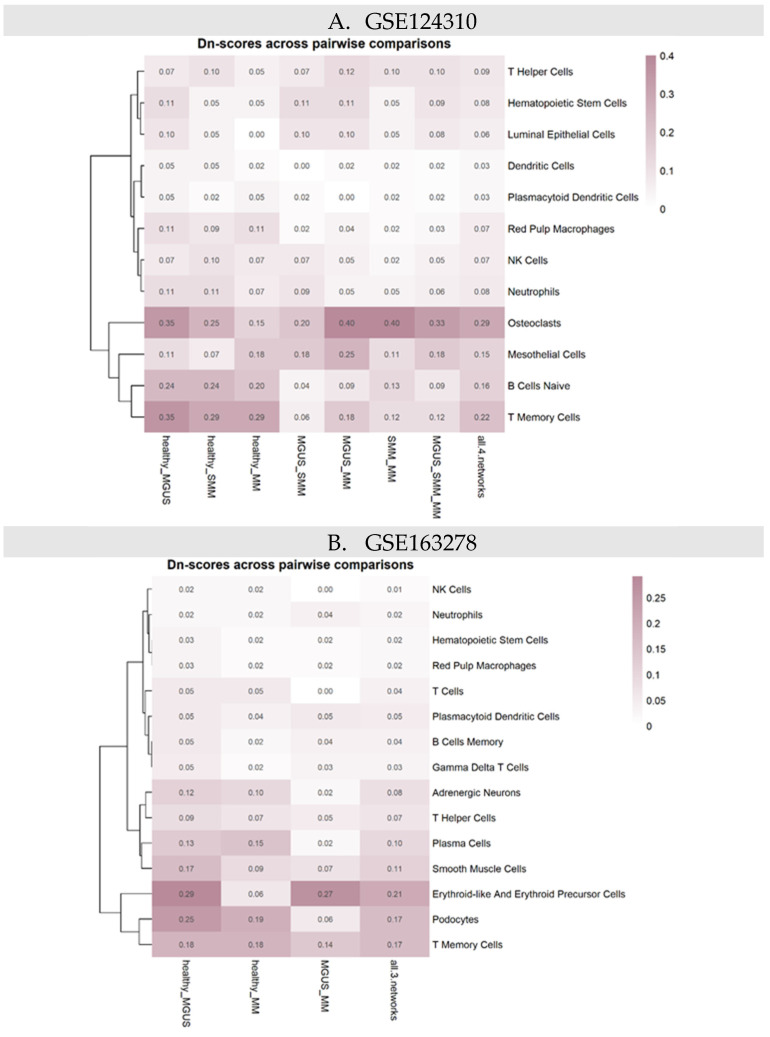

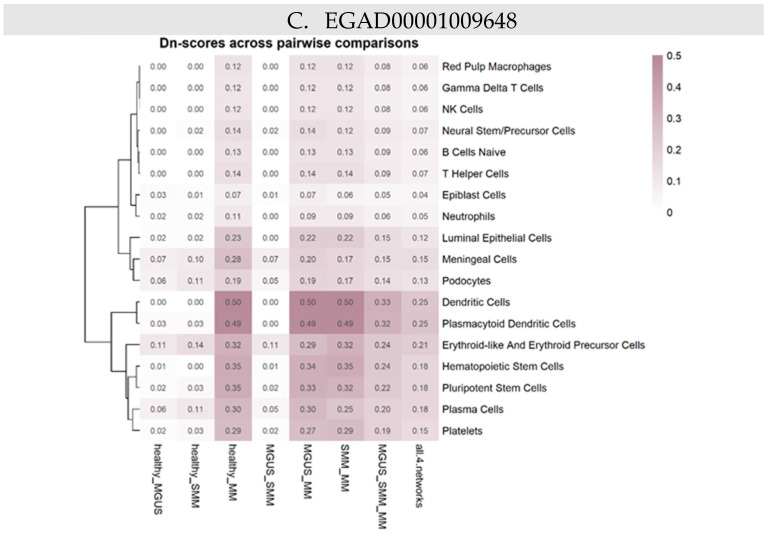
Degree-corrected Dn scoring between stage comparisons across datasets. (**A**–**C**) Heatmaps displaying Dn scores (degree-corrected rewiring metrics) for each cell type across pairwise or multi-condition network comparisons in the GSE124310, GSE163278, and EGAD00001009648 datasets, respectively. Rows represent cell types and columns correspond to comparison groups, with colour intensity reflecting the magnitude of the Dn score. Higher Dn scores indicate greater network rewiring and changes in cell-to-cell communication patterns between conditions, highlighting cell populations that are most dynamically altered across disease progression.

Changes in CCC are significant since they may play a crucial role in the progression of MM. With DyNet, we identified rewired nodes between pairwise and multiple network comparisons. Rewired nodes are those whose connections change across comparisons, in terms of changes in interacting neighbours and/or edge weights [17]. In the GSE124310 dataset, naïve B cells and T memory cells displayed high Dn scores in disease-stage networks when compared to the healthy reference network (Figure 4A). Notably, osteoclasts exhibited strong rewiring in progressive comparisons, particularly in MGUS with MM and SMM with MM comparisons, suggesting that osteoclast-associated communication undergoes major remodelling during transition to active MM.

In the GSE163278 dataset, T memory cells also emerged as highly rewired, with elevated Dn scores in the healthy versus MGUS and healthy versus MM comparisons (Figure 4B). In addition, erythroid-like cells and erythroid precursor cells demonstrated high rewiring, particularly in the healthy-MGUS and MGUS-MM comparisons. This suggests that, beyond immune remodelling, erythroid-associated communication programs may undergo significant restructuring both at early disease onset and during progression toward MM in this cohort.

In the EGAD00001009648 dataset, rewiring was most pronounced in antigen-presenting compartments. Specifically, DCs and pDCs exhibited consistently high Dn scores in comparisons involving the MM stage (healthy-MM, MGUS-MM, and SMM-MM) (Figure 4C). This indicates that DC- and pDC-mediated communication is particularly altered in the active MM stage, consistent with substantial disruption of immune surveillance mechanisms during MM progression. These results agree with the CCC networks and CytoHubba findings since the connections in DCs and pDCs were lost altogether in MM (Figure 2C), also assigned with NA in CytoHubba metrics (Figure 3C). 

### 2.4. JUN in Neutrophils Governs Downstream Targets, Suggesting a Role in MGUS-to-MM Disease Progression

**Figure 5:**
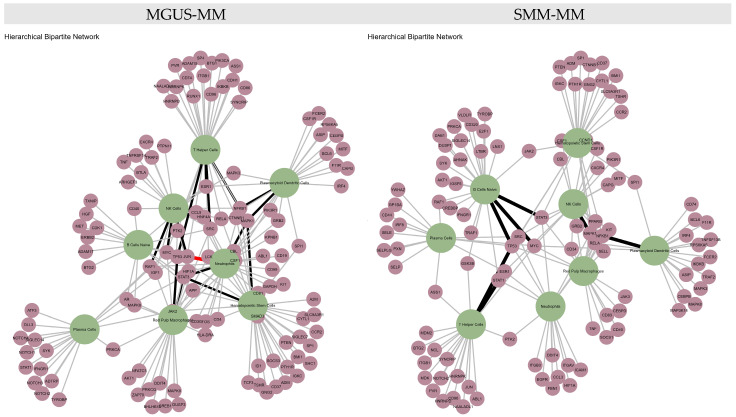
Common transcription factors across cell types of interest between the three datasets. MGUS-MM comparison across the GSE124310, GSE163278, and EGAD00001009648 datasets. SMM-MM comparison in the GSE124310 and EGAD00001009648 datasets. Purple nodes in the networks represent transcription factors connected to the cell types of interest (green nodes). Edge colours indicate the degree of conservation of transcription factor–cell-type associations across datasets: red edges denote transcription factors shared across all three datasets, black edges indicate transcription factors shared between two datasets, and grey edges represent transcription factors identified in a single dataset.

**Figure 6:**
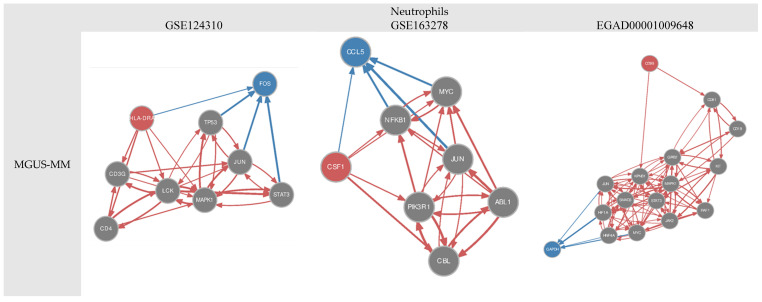

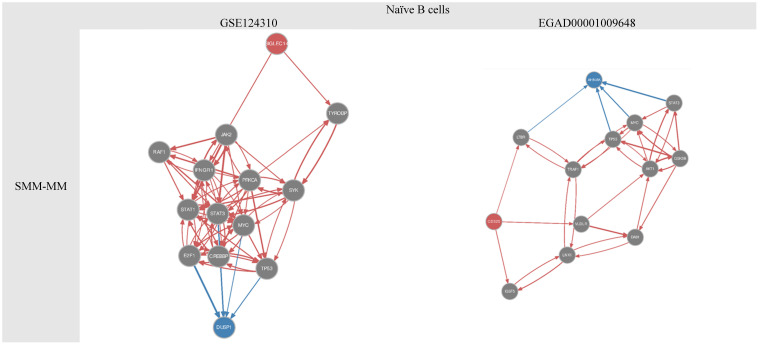
Ligand-to-target gene signalling pathways in progressive comparisons (MGUS-MM and SMM-MM). Ligand-mediated regulatory pathways inferred from MGUS-MM comparisons across the GSE124310, GSE163278, and EGAD00001009648 datasets, and from SMM-MM comparisons in the GSE124310 and EGAD00001009648 datasets. Analyses are shown for neutrophils and naïve B cells. The direction of the arrows indicates the information flow between molecules. The red colour suggests the upstream flow from the ligand and towards the TFs. The blue-coloured arrows suggest the downstream flow from the “final” TF towards the downstream target gene.

In the context of MM progression, we investigated common key factors across the three datasets that may underlie the transition from MGUS and SMM to MM. In the MGUS-MM, the transcription factor (TF) *JUN* was highlighted as a common player expressed in neutrophils across the three datasets (Figure 5A) (red edge). *JUN* further regulates distinct downstream top first target genes (Table 1). Information flow networks from the sender cell expressing ligand to the target gene in the receiver cell are shown in Figure 6. Additional information is given in Supplementary Figure S2A–C. Moreover, *ESR1*, *MAPK1*, *SRC*, *TP53*, *MYC*, *RAF1*, *STAT3*, and *NFKB1* are intermediate TFs common to the two datasets in cell types such as T helper cells, NK cells, HSCs, pDCS, and RPMs (black edges).

Likewise, for the SMM to MM stage comparison, *ESR1*, *TP53*, *GRB2*, *MAPK1*, *MYC*, and *STAT3* are downstream TFs common in T helper cells, NK cells, pDCs, and naïve B cells in the GSE124310 and EGAD00001009648 datasets (Figure 5B and Table 1). Naïve B cells were shown to be crucial cells for the progression of SMM to MM, with common TFs that further regulate downstream target genes. For instance, *MYC*, *STAT3*, and *TP53* genes were found as key factors in the signalling path in naïve B cells and regulate the expression of *DUSP1* and *AHNAK* in GSE124310 and EGAD00001009648 datasets, respectively (Figure 6 and Supplementary Figure S2D,E).

## 3. Discussion

**Figure 7:**
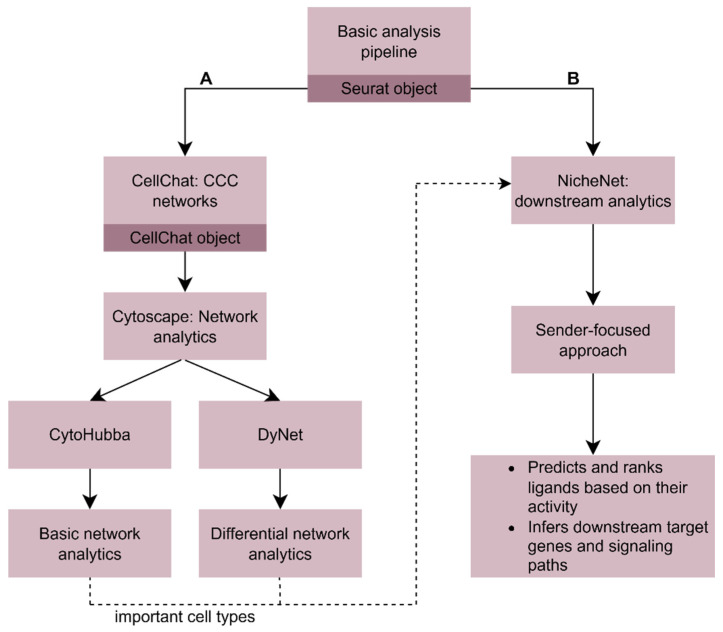
Overview of the computational workflow for cell-to-cell communication and downstream network analytics. Basic single-cell analytics were performed with Seurat and unique objects were created. Two complementary analytical routes were followed. (**A**) CCC networks were inferred using CellChat (version 2.1), by scRANK’s built-in function (version 1.0.0), and the resulting networks were exported to Cytoscape (version 3.10.2) for network-based analyses. Basic network analytics were performed using CytoHubba (version 0.1) to define key cell types, while network rewiring across conditions was assessed using DyNet (version 1.0.0) to identify rewired nodes. (**B**) In parallel, NicheNet (version 2.2.0) was applied in a sender-focused framework to predict and rank active ligands and infer downstream target genes and signalling pathways in receiver cells. The dashed arrow indicates the conceptual link between CCC-derived important cell types and NicheNet (version 2.2.0) downstream analysis.

In this work, we investigated how CCC changes in response to MM progression and identified cell nodes and downstream targets that may be affected. We were interested in the progression of the disease; therefore, we focused on MGUS-MM and SMM-MM progression comparisons. We developed a composite pipeline that incorporates public computational frameworks for the reconstruction and analysis of CCC networks, inferred from scRNAseq data. Two different yet overlapping routes of research were the backbone of the project (Figure 7). Network reconstruction with the scRANK’s built-in CellChat function was executed for the systematic analysis and visualisation of CCC networks. The emergence of candidate interactions and their rewiring provides a novel approach to understanding the biology of the disease.

Three publicly available scRNAseq datasets were analysed separately following the same analysis pipeline. While integrating datasets into a single framework could increase statistical power and enable direct cross-dataset comparisons, the heterogeneity in experimental design, sample composition, and CD138-selection strategies could pose challenges for reliable harmonization. By analysing each dataset independently, we aimed to minimize batch-driven artifacts and preserve biologically meaningful variation. Notably, hematopoietic lineage populations consistently emerged as prominent nodes. Therefore, we emphasized reproducible and consistent CCC patterns, robust across cohorts rather than driven by dataset-specific artifacts.

For the identification of key cellular compartments within the inferred CCC networks, weighted edge lists were analysed with CytoHubba in Cytoscape. Degree centrality, betweenness centrality, and bottleneck metrics were computed to highlight cell types occupying topologically important cell nodes in the networks. The EGAD00001009648 dataset displayed overall higher degree centrality values compared to the other two datasets, likely reflecting its larger number of cell types and consequently increased network complexity. Across all datasets, DCs, pDCs, HSCs, and RPMs consistently ranked among the most highly connected nodes, suggesting that these populations act as cell types with major communication in the bone marrow TME.

Studies on *S. cerevisiae* correlate the degree of a protein with its essentiality, suggesting that highly connected nodes represent molecules that tend to be more important [18,19]. Moreover, high connectivity in human and mouse PPI networks has been linked to functional importance and essentiality through the well-established “centrality–lethality” principle. This suggests that highly connected proteins are more likely to be essential due to their involvement in multiple interactions [20]. However, the direct translation of this principle to CCC networks and interactions between cell types rather than proteins should be interpreted with caution.

This concept provides a useful analogy: highly connected cell nodes may represent cellular compartments that coordinate multiple signalling relationships and exert broad influence over microenvironmental communication. In this context, the consistent high degree centrality of pDCs in all three datasets is notable. While the exact mechanistic role of pDCs in MM biology remains incompletely defined, previous studies have shown that altered pDC frequencies in the bone marrow of MGUS and MM individuals are associated with disease control and treatment outcome [21]. Moreover, pDCs trigger MM cell growth and prolong survival by activating the growth signalling kinase ERK [22].

In addition to highly connected nodes, we examined bottlenecks, which are defined as nodes that participate in many shortest paths and are therefore predicted to act as crucial intermediates supporting overall network connectivity [23]. Moreover, it is known that bottlenecks can be defined based on the high betweenness centrality of the node in the network [24]. As expected, bottlenecks largely overlapped with cell types exhibiting high betweenness centrality. Across datasets, pDCs, RPMs, HSCs, and B cells (naïve and memory) were recurrently highlighted as bottlenecks in MGUS networks, suggesting that early disease stages may depend on a limited set of immune and myeloid populations that structurally connect multiple cellular compartments. In later stages, bottleneck roles shifted toward lymphocyte-centred connectors, with naïve B cells emerging as bottlenecks in SMM and T helper cells in MM, consistent with progressive reorganization of communication routes toward adaptive immune and macrophage-associated signalling as disease advances.

CCC rewiring is increasingly recognised as a driving force in disease progression, where dynamic changes in intercellular communication support cancer’s hallmarks [25]. It was also shown in neurodegeneration that the miscommunication of cells and perturbations can enhance the progression of the disease [26]. Similarly, altered CCC in our data suggests a role in MM’s progression. From our analyses, memory T cells and naïve B cells exhibit high Dn scores, indicating node rewiring and variation between disease and healthy networks in the GSE124310 dataset. Moreover, DCs and pDCs in the MGUS-MM and SMM-MM progressive comparisons, in the EGAD00001009648 dataset, exhibited a high Dn score, highlighting their potential role in disease progression [17]. Additionally, it is known from the literature that DCs accumulate in MM and protect malignant plasma cells from killing by T helper cells [27].

Based on a “dataset union” NicheNet approach on the eight cell types of interest, neutrophils commonly express the TF *JUN* across the three datasets in the MGUS-MM progressing stage comparison. The *HLA-DRA* ligand is a component of the Major Histocompatibility Complex (MHC) class II molecule and plays a critical role in immune surveillance; its expression levels change during the progression from MGUS to active MM [28]. *HLA-DRA* expression profiles undergo shifts in myeloid lineage cells, particularly within the bone marrow microenvironment, facilitating immune evasion and transition from MGUS to MM, highlighting it as a key indicator of disease progression [29]. Moreover, the literature supports that MM individuals exhibit neutrophil dysfunction, making them more susceptible to bacterial infections [30].

Importantly, *JUN* across the three datasets regulates distinct downstream target genes in neutrophils: *FOS*, *CCL5*, and *GAPDH*. *FOS* is a proto-oncogene, a subunit of AP-1, a member of the AP-1 family genes, similar to *JUN*. Although these genes are ranked as the highest expressed in malignant plasma cells, they do not give any prognostic information [31]. In agreement with the literature, JUN and FOS are implicated in plasma cell differentiation and MM pathogenesis [32]. Beyond its role in cancer, JUN also has a well-established role in normal haematopoiesis through the PU.1–c-JUN–GATA-1 regulatory axis. In this context, c-JUN cooperates with PU.1 (Purine-rich box 1) to promote myelopoiesis, while it is an antagonist of GATA-1-driven erythroid differentiation [33]. The balance of these TFs has been shown to determine whether blood cells become myeloid or erythroid, showing that JUN activity depends strongly on cellular context and lineage rather than being purely oncogenic. Additionally, FOS is crucial for disease progression and drug resistance in MM [34]. Moreover, c-FOS expression was significantly higher in MM cells and was associated with poor prognosis in patients [35]. CCL5 (C-C motif chemokine ligand 5) is a chemokine that, in malignant cells, is used to evade immune cell or medication-induced apoptosis, escape the bone marrow, and escalate bone lesions. Du et al. suggest that anti-tumour chemokines or blockage of pro-tumour chemokines is a promising therapeutic strategy in MM [36]. An unexpected observation was the appearance of *GAPDH*, among the predicted downstream target genes, in the neutrophil-centred signalling analysis. Although *GAPDH* is commonly treated as a housekeeping and metabolic gene, its expression can vary across activation states and may reflect broader metabolic and transcriptional reprogramming in cancer-associated immune microenvironments. In the context of MM, *GAPDH* reinforces the pro-tumour metabolic role and also makes the MM prognosis poorer [37].

Other *TFs (ESR1, MAPK1, SRC, TP53, MYC, RAF1, STAT3, NFKB1)* common to two out of the three datasets were shown to be key drivers for the progression of MGUS to MM, since they participate in several pro-survival and proliferative signalling pathways. For instance, MAPK1 and RAF1 are members of the MAPK cascade that is frequently activated in MM and drive cell proliferation and survival of malignant cells [38]. NFKB1 is implicated in the NF-κB pathway, whose abnormal activation promotes malignant plasma cell growth and survival [39]. Collectively, the TFs revealed by our analyses converge on core oncogenic signalling pathways such as MAPK, JAK/STAT, NF-κB, and MYC, which cooperate to promote cancer hallmarks such as malignant cell survival and proliferation, resulting in the progression from MGUS to MM [38,39,40].

For the progression of SMM to MM, our results highlighted *ESR1* and *TP53* TFs in T helper cells, *GRB2*, and *MAPK1* in NK cells and *MYC*, *STAT3*, and *TP53* TFs in naive B cells, common across the two datasets, including SMM samples. These TFs align along conserved signalling cascades, especially the p53 checkpoint. MAPK/ERK activation and the STAT3-mediated survival pathway coordinate immune dysregulation and proliferative reprogramming to support MM pathophysiology. Collectively, immature naïve B cells, with the assistance of T helper cells that release cytokines, differentiate into plasma cells [41]. The primary affected cell type in MM is the malignant plasma cells that proliferate uncontrollably in the bone marrow, resulting in various complications [30,42]. In normal conditions, NK cells target and kill malignant cells. However, in MM, they malfunction, making them unable to combat the malignant plasma cells. Interestingly, NK cells are research targets for cancer immunotherapy [41]. Collectively, these cell types are crucial for the establishment of blood cells in normal conditions, yet their aberrant CCC can drive MM progression.

This hypothesis-generating framing lays the foundation for future investigations for targeting aberrant CCC. Moreover, it underscores their potential as a promising therapeutic strategy in the management of MM. Although immune checkpoint inhibitor (ICI) therapies targeting the Programmed Death 1 (PD-1/PD-L1) axis have transformed treatment in several solid tumours, their efficacy in MM has been limited [43]. PD-L1 is frequently upregulated on malignant plasma cells and immune cells in the MM bone marrow microenvironment, contributing to T-cell inhibition and tumour immune escape [44,45]. Treatment studies on PD-1/PD-L1 blockade have been revolutionized; however, in MM, discordant results have been reported by several teams [46]. A combinatorial treatment of ICI and immunomodulatory drugs for MM suggests a logical treatment [46]. Along with ICIs, there is a class of agents that modify the immune system to either suppress or enhance its activity: called immunomodulatory drugs (IMiDs). Clinical use of IMiDs has significantly improved long-term survival and quality of life of MM patients [47]. Moreover, proteasome inhibitors (PIs) are anti-cancer drugs that block the action of proteasome, resulting in multiple downstream effects, including the inhibition of NF-κB signalling, protein build-up, leading to tumour cell death via apoptosis [48]. These results highlight the complexity of the immunosuppressive microenvironment in MM and suggest that while immune regulation is clearly altered, effective therapeutic modulation of checkpoint pathways likely requires rational combinations and deeper biological understanding.

Despite the strengths of this study, several limitations should be considered when interpreting the findings. A limitation of this study is the limited representation of bone-resident cell populations, such as osteoclasts, due to the CD138− based selection of the analysed datasets. In addition, the restriction to CD138 populations inherently excludes malignant plasma cells, which are central drivers of MM biology. Among our goals in this work was to study intercellular communication patterns in disease progression; we therefore focused on CD138− populations. The inferred CCC networks should be interpreted as microenvironment-specific, reflecting interactions among non-malignant immune and stromal components rather than the full tumour ecosystem. In this work, our findings are microenvironment-specific, rather than representative of the full disease landscape. As a result, key TME interactions involving malignant plasma cells are not captured in the present analysis. Future studies leveraging CD138+ cell types will be informative to fully dissect tumour, bone-resident populations and MM remodelling.

Another limitation of this study is that downstream analyses prioritize housekeeping genes such as *GAPDH*. The identification of *GAPDH* as a downstream target should not be interpreted as a specific regulatory endpoint, but rather as a proxy for broader transcriptional or metabolic shifts. This highlights the need for a cautious interpretation of predicted ligand-to-target links and reinforces the importance of validation strategies.

In this study, the Dn score was computed using the DyNet framework, which deterministically quantifies changes in network composition across stage-specific networks. Importantly, CCC networks are biologically constrained, as edges derived from LR pairs supported by the scRNAseq data. This is a straightforward calculation rather than an inference model needing statistical validation. Nevertheless, the choice of the algorithm and functions used for network differentiation may alter the final calculations. To mitigate this limitation, our analysis focuses on relative, stage-specific differences in networks across disease progression, rather than on the absolute significance of individual rewiring scores.

Experimental validation of inferred LR pair-mediated interactions and downstream signalling programs is beyond the scope of this study. Interpretations related to disease mechanisms or therapeutic targeting should be considered as hypothesis-generating rather than definitive conclusions. Therefore, our findings should be interpreted as computationally derived hypotheses that prioritize candidate CCC axes and regulatory programs for future validation. The predicted interactions and network features highlight potential regulatory axes within the bone marrow TME that warrant further investigation through experimental and functional validation. Follow-up, spatial transcriptomics (e.g., in situ sequencing) could be applied to bone marrow samples from a reference condition (healthy or early-stage disease) and compared against later-stage samples, enabling the evaluation of gene expression programs in their native tissue context and resolving the spatial localization of interacting cell populations. In addition, co-culture assays could be used to functionally test predicted interactions between key immune and stromal compartments identified by network topology and rewiring analyses, including naïve B cells, memory T cells, DCs, pDCs, HSCs, RPMs, and NK cells. Future studies integrating perturbation experiments and multi-modal data will be essential to confirm the biological relevance and therapeutic potential of the identified candidates. Finally, perturbation experiments could directly assess the regulatory structure of inferred signalling pathways in neutrophils during the progression of MGUS to active MM, which could be perturbed (e.g., knockdown/knockout or pharmacological inhibition) and then downstream effects such as survival, proliferation, and apoptosis measured.

## 4. Materials and Methods

### 4.1. R Packages, Tools, and Code Availability

Seurat v5 was used for basic, standard scRNAseq analytics [49]. Single Cell Ranking Analysis Toolkit (scRANK) version 1.0.0 was mainly used for the reconstruction of CCC networks by the integrated “runCellChat” function [50]. The publicly available plug-ins CytoHubba (version 0.1) and DyNet (version 1.0.0) were downloaded in Cytoscape (version 3.10.2) [8,17,23]. NicheNet R package version 2.2.0 was used for the identification of downstream target genes in the receiver cells, influenced by sender-expressed ligands [2]. The packages and corresponding documentation were downloaded and installed through GitHub (https://github.com/). The flowchart in Figure 7 shows the pipeline of the tools that were used in this study, following two different routes.

The full analysis workflow used in this work, including scripts and documentation for CCC inference and downstream NicheNet analyses, has been made publicly available in a dedicated GitHub repository: https://github.com/eleninic/CCC_analysis, accessed on 2 April 2026.

### 4.2. Dataset Collection and Implementation

A systematic review in public repositories was performed to retrieve scRNAseq datasets with human bone marrow samples from healthy and MM-diseased individuals. The three publicly available datasets, along with additional information, are summarised in Supplementary Table S4. This paper analyses existing, publicly available data, accessible on the Gene Expression Omnibus (GEO) database (https://www.ncbi.nlm.nih.gov/geo/) (accessed on 8 January 2024) with accession numbers GSE124310 and GSE163278 and the European Genome–Phenome Archive (EGA) database (https://ega-archive.org/) with the accession number EGAD00001009648 (accessed on 8 January 2024) [51]. The GSE124310 dataset was generated in a scRNAseq study profiling bone marrow immune cells from healthy donors and MGUS, SMM, and MM individuals to map how the immune microenvironment changes during disease progression [52]. The GSE163278 dataset was also deposited in the GEO database, in a study where cells from bone marrow samples of healthy individuals and patients (MGUS, MM) were profiled using single-cell technologies to compare cellular composition and states during disease progression [53]. The EGAD00001009648 dataset was generated via single-cell RNA and B-cell receptor sequencing to profile tumour precursor and MM cells from patients with MGUS, SMM, and MM alongside normal donors to characterise clonal and transcriptional evolution [54]. The raw sequencing data processing of this dataset is described in the corresponding section in the Supplementary Materials.

The three publicly available scRNAseq datasets analysed in this study were different in sample size, cell-type composition, CD138 composition, and the disease stages that they include (Supplementary Table S4). This heterogeneity enabled the evaluation of the pipeline across distinct datasets. The GSE124310 and EGAD00001009648 datasets included samples from healthy donors, MGUS, SMM, and MM, while the GSE163278 dataset did not include SMM samples. Despite these differences, the pipeline consistently identified biologically meaningful interactions across datasets, demonstrating its applicability irrespective of sample size, cell type diversity, or disease-stage coverage.

### 4.3. CD138 MM Marker Cell Sorting

Three publicly available scRNAseq datasets were processed and analysed separately. All the datasets were analysed following the same protocol. Performing independent analysis of each dataset enabled the assessment of robustness and reproducibility of the identified communication patterns across distinct cohorts.

To focus on biologically relevant interactions in the bone marrow TME, we retained cells of the CD138− compartment, enabling the analysis of CCC patterns in both healthy and disease samples. GSE124310 and GSE163278 datasets included only CD138− samples, whereas EGAD00001009648 includes both CD138-positive and -negative samples. CD138 is an MM plasma cell marker indicating cancer cells in the TME, whereas CD138− cells are non-cancerous cells from the bone marrow [55] (Supplementary Table S4).

During data pre-processing, samples originating from CD138+ fractions were excluded, and quality control filtering ensured that no CD138+ plasma or other cells were present in the final analysed datasets. Although the EGAD00001009648 dataset contains both CD138+ and CD138− sorted samples, only CD138− samples were selected for inclusion in this study. As a result, all inferred CCC networks reflect interactions among immune and stromal components of the TME and do not include direct interactions involving malignant CD138+ plasma cells. Noting that in the EGAD00001009648 dataset, 1 out of 14 negative MGUS samples was not used due to low cell count. Also, 1 out of 3 MM samples was not used since the individual was under RRMM (Relapsed/Refractory Multiple Myeloma) treatment (Supplementary Table S4).

### 4.4. Basic Pre-Processing of scRNAseq Data

Basic dataset analysis was performed following the Seurat v5 pipeline https://satijalab.org/seurat/ (accessed on 2 April 2026) [49]. Each dataset was analysed separately and the samples were separated according to their condition.

For each sample, a Seurat object was created by filtering those with at least 3 cells and 200 features. All Seurat objects were then merged and unique cell IDs were assigned to each sample according to their disease stage. After normalisation, feature selection, and data scaling, principal component analysis (PCA) was performed. Before integrating the data, we proceeded with neighbour-finding and clustering on the unintegrated data.

For visualisation purposes, Uniform Manifold Approximation and Projection (UMAP) was used. Anchor-based canonical correlation analysis (CCA) was selected for the integration of PCA analyses. This approach identifies shared correlation structures among datasets. The RNA layers of all the samples were then merged and, like the unintegrated analysis, neighbours and clusters were found.

After clustering, the marker genes for all the clusters were calculated using the Wilcoxon Rank Sum test, which identifies differentially expressed genes between groups of cells. By keeping the top 100 marker genes according to their log2FC ranking, using the enrichR package in R (version 3.4), PanglaoDB (version 2021) (https://panglaodb.se/, accessed on 8 November 2024) was used to assign cell types to their related clusters. Clusters assigned to the same cell type were merged and those that were not assigned to a cell type were merged into the closest cell type according to the UMAP.

### 4.5. CCC Network Reconstruction with CellChat

For the reconstruction of CCC networks, a tool previously developed in our lab (scRANK, version 1.0.0), based on the “Inference and analysis of cell–cell communication using CellChat” vignette (version 2.1) [50], was used (https://github.com/aoulas/scRANK, accessed on 2 April 2026). The LR interaction database was set for human and the communication probability was computed using “computeCommunProb” with the default parameter type = “TriMean” [7]. This function calculates the number of inferred LR pairs specially producing fewer but stronger interactions mediating CCC. Communication was filtered with the minimum number of cells required for the CCC, set to 10. The communication probability of signalling pathways (“computeCommunProbPathway”) was calculated to associate LR pairs with downstream signalling pathways [7].

A unique, weighted and directed CCC network was created for each stage of MM and healthy controls, visualising the number of interactions/counts (edges) between two cell types (nodes) (see Section 2). The size of each node is proportional to the cell population and the edge width corresponds to the number of LP pair-mediated interactions between the two nodes. The CCC networks are weighted, suggesting the communication score of each interaction, that is, the LR pairs mediate each specific intercellular interaction between two cell types [7]. Moreover, the networks are directed, indicating the information flow from the sender cell expressing the ligand towards the receptor expressed on the surface of the receiver cell. Normalisation of cell proportions was performed by dividing the number of cells in a specific condition by the total number of cells in the same condition. Additionally, autocrine communication, mediated by self-loops, is also depicted since it suggests interactions within the same cell population.

### 4.6. Basic Network Analytics Based on Network Topology—Prioritisation of Key Cell Types with CytoHubba

Edge lists of CCC networks were the input in Cytoscape for CytoHubba analytics, which predicts and explores significant cell nodes based on network topology. It calculates 11 different topology metrics and identifies the most important cell nodes. Therefore, basic network analyses were performed to identify important nodes in the networks by calculating degree centrality, betweenness centrality, and bottlenecks [23]. Degree centrality is the number of edges connected to a node; betweenness centrality is how often a node acts as a bridge along the shortest paths between two nodes; and bottlenecks suggest nodes that are critical connectors in the network topology [23].

### 4.7. Differential Network Rewiring Analysis in Response to MM Progression with DyNet

Weighted edge lists for each CCC condition network, per dataset, were the input in Cytoscape for differential network analysis with the DyNet plug-in. DyNet visualises node changes in response to the presence or absence of cell nodes across the networks. With DyNet Analyser, networks were treated as directed and the most varying nodes in the aspect of “DyNet REWIRING” were highlighted by performing pairwise and multiple network comparisons: healthy-MGUS, healthy-SMM, healthy-MM, MGUS-SMM, MGUS-MM, SMM-MM, and MGUS-SMM-MM (when SMM was present).

Data were normalised using the average of non-zero values. A degree corrected rewiring score (Dn score: dynamic neighbourhoods score) was assigned, capturing the change in node connectivity across networks, based on a statistical formula [17]. A high Dn score indicates high variance and increased rewiring, whereas a low Dn score indicates low variance between the networks under comparison [17]. A Dn score equal to zero indicates stable interactions since the number of connections between the networks’ nodes remains unchanged [17].

### 4.8. NicheNet Downstream Analyses

#### 4.8.1. Identification of Cell Types of Interest

In subsequent analyses, we aimed to study and understand the progression of MGUS and SMM to MM. Therefore, we focused on the stage comparisons between MGUS-MM and SMM-MM. We conducted a thorough literature review, considering the six common cell types identified in the three datasets, as well as additional key cell types highlighted in our prior network analyses. Based on this, we identified HSCs, naïve B cells, T helper cells, plasma cells, NK cells, neutrophils, pDCs, and RPMs as the most relevant cell types associated with MM.

#### 4.8.2. Ligand Prioritisation in Sender Cells and Identification of Downstream Target Genes in Receiver Cell Types

To investigate ligands from the sender cells that may influence the gene expression of downstream target genes in receiver cells, pairwise progression comparisons were performed (MGUS-MM and SMM-MM). A custom R script, based on the “Perform NicheNet analysis starting from Seurat object” vignette (https://github.com/saeyslab/nichenetr/blob/master/vignettes/seurat_wrapper.md, accessed on 2 April 2026), was executed. The prior ligand–target matrix, ligand–receptor network, and weighted signalling networks were obtained from the NicheNet package (version 2.2.0) and used without modification.

Following the function “nichenet_seuratobj_aggregate” of the sender-focused approach, Seurat Objects of each dataset were loaded and updated using the “UpdateSeuratObject” function. For each pairwise comparison, a reference condition and a condition of interest were defined (the earliest stage was considered the control). Differential expression between the two conditions was calculated by default with the standard Seurat Wilcoxon test. Genes of interest in receiver cells were defined as discussed in the previous section. The background gene set was defined as all genes expressed in the receiver cell population. The number of top-ranked ligands was set to 30 (“top_*n*_ligands” = 30) and the number of target genes to consider per ligand was set to 200 (“top_*n*_targets” = 200). Ligands were ranked based on their activity scores, computed as Pearson’s correlation between predicted target gene regulation and the observed differential expression profile in receiver cells, as implemented in NicheNet. Furthermore, we retained ligands and receptors that were expressed in at least 5% of cells in one cluster (“expression_pct = 0.05”). Candidate ligands were required to be expressed in sender cells and to have at least one corresponding receptor expressed in receiver cells, using the same expression threshold. The senders were set to ‘all’ and the receivers were set to the unique cell types in each object. All parameters and analysis steps are provided in the accompanying GitHub repository (https://github.com/eleninic/CCC_analysis/blob/main/NicheNet.r, accessed on 2 April 2026) to ensure reproducibility.

#### 4.8.3. Inference of Ligand-to-Target Links with High Potential to Be Regulated by Prioritised Ligands

For further understanding of crucial players, such as top-ranked ligands, TFs, and downstream target genes of interest, we ran the vignette “Inferring ligand-to-target signalling paths” of NicheNet that can be found here: https://github.com/saeyslab/nichenetr/blob/master/vignettes/ligand_target_signaling_path.md (accessed on 2 April 2026). NicheNet infers regulatory relationships between ligands, TFs, and target genes [2]. Ligand–target links were prioritized based on regulatory potential scores derived from the NicheNet model.

Players in the path between ligands and target genes were considered important signal mediators. Normalisation of edge weights was performed for accurate comparisons with the parameter “minmax_scaling=TRUE”. Each top first ligand was further analysed with its downstream top first target genes to identify TFs that play a role in the signalling pathway downstream. TFs that were common in three and two datasets for MGUS-MM and SMM-MM pairwise comparisons, respectively, were further studied for their downstream target genes. Common TFs were defined based on their presence across datasets using intersection criteria (i.e., shared across 3 or 2 datasets, depending on the comparison).

## 5. Conclusions

To conclude, with this project, we established a unique reproducible pipeline incorporating well-known, publicly available computational tools to study CCC, altered interactions, and downstream target genes in the progression of MM. This combined hypothesis-generating framework provides novel biological insights into regulatory mechanisms and intercellular communication patterns that cannot be captured by single-tool analyses, emphasizing the added value of a composite and cross-dataset framework. Our pipeline thus offers a generalizable strategy for dissecting the dynamics of the tumour microenvironment in MM and may serve as a blueprint for similar studies in other haematological malignancies. However, along with genetic factors, tumour exposome, with all the environmental exposures that interact with the tumour and its microenvironment, shape cancer’s pathophysiology, disease initiation, progression, and response to treatment. Collectively, CCC rewiring in crosstalk with tumour exposome will provide valuable insights into mechanisms that drive MM.

Along with our future directions, we aim to leverage in silico drug repurposing approaches to target aberrant CCC for the management of MM, with prior knowledge-based repurposing at the single-cell-level resolution. This strategy has the potential to accelerate the discovery of candidate therapeutic approaches for MM.

## Figures and Tables

**Table 1:** Downstream target genes influenced by the top-ranked common transcription factors across the datasets for the progressing comparisons: MGUS-MM and SMM-MM.

	Transcription Factor	Cell Types	Downstream Target Genes		
			GSE124310	GSE163278	EGAD00001009648
MGUS-MM	*JUN*	Neutrophils	*FOS*	*CCL5*	*GAPDH*
		NK cells	*CCL5*	*ARFGEF3*	*-*
	*ESR1*	T helper cells	*-*	*BTG1*	*ASS1*
	*MAPK1*	HSCs	*-*	*A2M*	*CD37*
		Neutrophils	*FOS*	*-*	*GAPDH*
		pDCs	*-*	*CAPG*	
		T helper cells	*CD74*	*BTG1*	*-*
	*MYC*	NK cells	*CCL5*	*ARFGEF3*	*CXCR4*
		Neutrophils	*-*	*CCL5*	*GAPDH*
	*NFKB1*	pDCs	*-*	*CAPG*	*FCER2*
	*RAF1*	NK cells	*-*	*ARFGEF3*	*CXCR4*
	*SRC*	T helper cells	*CD74*	*-*	*ASS1*
	*STAT3*	HSCs	*IGKC*	*A2M*	*-*
		Neutrophils	*FOS*	*-*	*GAPDH*
		RPMS	*BHLHE40*	*FOS*	*-*
	*TP53*	RPMs	*-*	*FOS*	*DDIT4*
		T helper cells	*-*	*BTG1*	*ASS1*
SMM-MM	*ESR1*	T helper cells	*BTG2*	*-*	*ASS1*
	*GRB2*	NK cells	*CAPG*	*-*	*CXCR4*
	*MAPK1*	NK cells	*CAPG*	*-*	*CXCR4*
	*MYC*	Naïve B cells	*DUSP1*	*-*	*AHNAK*
	*NFKB1*	pDCs	*CD74*	*-*	*FCER2*
	*STAT3*	Naïve B cells	*DUSP1*	*-*	*AHNAK*
	*TP53*	Naïve B cells	*DUSP1*	*-*	*AHNAK*
		T helper cells	*BTG2*	*-*	*ASS1*

## Data Availability

This study did not produce any new data. The three publicly available datasets are accessible at Gene Expression Omnibus (GEO) database (https://www.ncbi.nlm.nih.gov/geo/) with accession numbers GSE124310 and GSE163278 (accessed on 8 January 2024) and the European Genome–Phenome Archive (EGA) database (https://ega-archive.org/) with the accession number EGAD00001009648 (accessed on 8 January 2024). The full analysis workflow used in this work, including scripts and documentation for CCC inference and downstream NicheNet analyses, has been made publicly available in a dedicated GitHub repository: https://github.com/eleninic/CCC_analysis (accessed on 2 April 2026).

## Supplementary Materials

The following supporting information can be downloaded at: https://www.mdpi.com/article/10.3390/ijms27114986/s1. Reference [56] is cited in the Supplementary Materials.

## Abbreviations

The following abbreviations are used in this manuscript:

CCA	Canonical correlation analysis
CCC	Cell-to-cell communication
CCL5	C-C motif chemokine ligand 5
DCs	Dendritic cells
EGA	European Genome–Phenome Archive
GEO	Gene Expression Omnibus
HSCs	Hematopoietic stem cells
ICIs	Immune checkpoint inhibitor therapies
IMiDs	Immunomodulatory drugs
LR	Ligand–receptor
MGUS	Monoclonal gammopathies of uncertain significance
MHC	Major Histocompatibility Complex
MM	Multiple Myeloma
NKs	Natural killer cells
PCA	Principal component analysis
PD	Programmed death axis
pDCs	Plasmacytoid dendritic cells
PIs	Proteasome inhibitors
PU.1	Purine-rich box 1
RPMs	Red pulp macrophages
RRMM	Relapsed/Refractory Multiple Myeloma
scRANK	Single cell rankins analysis tool
scRNAseq	Single-cell RNA sequencing
SMM	Smouldering Multiple Myeloma
TF	Transcription factor
TME	Tumour microenvironment
UMAP	Uniform manifold approximation and progression
