# Supplementary material for: Network-Based Bioinformatics Reveal Microenvironment-Driven Cell-to-Cell Communication in the Progression of Multiple Myeloma
Source: Int J Mol Sci. 2026 May 30;27(11):4986. doi: 10.3390/ijms27114986 (PMC13256439; doi:10.3390/ijms27114986)
Supplement: Supplementary file 1 [file ijms-27-04986-s001.zip › ijms-4269026-supplementary.pdf]

Supplementary Materials:

EGAD00001009648 raw sequencing data processing

Raw sequencing data of the EGAD00001009648 dataset were processed using a custom Bash pipeline designed to automate data retrieval, conversion, and quantification of scRNAseq libraries. The pipeline takes as input a tab-delimited metadata file specifying sequencing file locations and accession identifiers.

For each line in the input file, entries containing the string "BCR" were excluded from downstream processing. Remaining lines were parsed to extract (i) EGA accession identifiers (matching the pattern EGAF0000*) and (ii) associated BAM file names. BAM file names were standardized by removing the prefix scs_5GEX_ to ensure compatibility with downstream tools.

Raw sequencing data were retrieved from the EGA using pyega3 with authenticated credentials, enabling parallelized downloads (20 concurrent connections). Following download, BAM files were converted to FASTQ format using bamtofastq with multithreading (20 threads). After successful conversion, the original BAM files were removed to conserve disk space.

FASTQ files were automatically located within the generated directory structure and used as input for gene expression quantification with Cell Ranger (10X Genomics) [1]. Transcript quantification was performed using the Cell Ranger Count pipeline with default parameters, specifying the GRCh38 human reference transcriptome (Cell Ranger reference version 3.0.0). Each dataset was processed independently, with unique run identifiers corresponding to the original EGA accession. All analyses were executed from a centralized working directory to ensure consistent file paths and reproducibility across samples.

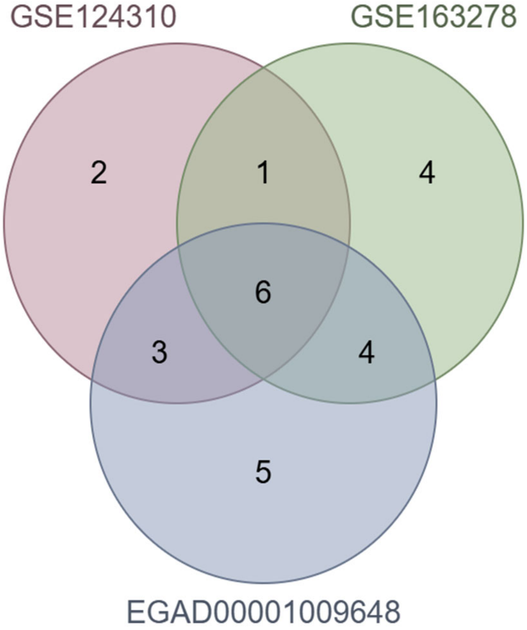

Supplementary Figure S1. Venn diagram showing the distribution of shared and unique cell types across datasets.

Six cell types were identified as common across all three datasets: Hematopoietic Stem Cells, NK Cells, Neutrophils, Plasmacytoid Dendritic Cells, Red Pulp Macrophages and T Helper Cells.

Supplementary Table S1. The occurrence of each cell type in the three datasets.

Across the three datasets, Hematopoietic Stem Cells, NK Cells, Neutrophils, Plasmacytoid Dendritic Cells, Red Pulp Macrophages and T Helper Cells are common. Remarkably, there are cell types unique per dataset too.

Cell Type	Occurrence	Dataset Present in
Hematopoietic Stem Cells	3	all
NK Cells	3	all
Neutrophils	3	all
Plasmacytoid Dendritic Cells	3	all
Red Pulp Macrophages	3	all
T Helper Cells	3	all
B Cells Naive	2	GSE124310, EGAD00001009648
Dendritic Cells	2	GSE124310, EGAD00001009648
Luminal Epithelial Cells	2	GSE124310, EGAD00001009648
Erythroid-like And Erythroid Precursor Cells	2	GSE163278, EGAD00001009648
Gamma Delta T Cells	2	GSE163278, EGAD00001009648
Plasma Cells	2	GSE163278, EGAD00001009648
Podocytes	2	GSE163278, EGAD00001009648
T Memory Cells	2	GSE124310, GSE163278
Mesothelial Cells	1	GSE124310
Osteoclasts	1	GSE124310
Adrenergic Neurons	1	GSE163278
B Cells Memory	1	GSE163278
Smooth Muscle Cells	1	GSE163278
T Cells	1	GSE163278
Epiblast Cells	1	EGAD00001009648

Meningeal Cells	1	EGAD00001009648
Neural Stem/Precursor Cells	1	EGAD00001009648
Platelets	1	EGAD00001009648
Pluripotent Stem Cells	1	EGAD00001009648

Supplementary Table S2. Non-normalized cell counts in the datasets (A. GSE124310, B. GSE163278, C. EGAD00001009648) in the progressive stages of the disease and healthy controls.

**GSE124310**	**HEALTHY**	**MGUS**	**SMM**	**MM**
B Cells Naive	400	90	312	480
Dendritic Cells	66	48	83	82
**Hematopoietic Stem Cells**	845	121	310	301
Luminal Epithelial Cells	480	41	138	248
Mesothelial Cells	85	13	135	1521
**Neutrophils**	447	353	474	571
**NK Cells**	1771	1210	3033	2670
Osteoclasts	555	149	454	968
**Plasmacytoid Dendritic Cells**	172	17	56	59
**Red Pulp Macrophages**	328	295	483	606
**T Helper Cells**	740	1024	2081	974
T Memory Cells	547	93	238	1025
**GSE163278**	**HEALTHY**	**MGUS**	**SMM**	**MM**
Adrenergic Neurons	1059	3647	NA	1381
B Cells Memory	1377	3291	NA	1058
Erythroid-like And Erythroid Precursor Cells	572	2390	NA	1337
Gamma Delta T Cells	403	531	NA	748
**Hematopoietic Stem Cells**	176	441	NA	730
**Neutrophils**	1574	1501	NA	1882
**NK Cells**	3038	4568	NA	4671
Plasma Cells	45	404	NA	258
**Plasmacytoid Dendritic Cells**	350	575	NA	491
Podocytes	804	1953	NA	1552
**Red Pulp Macrophages**	167	229	NA	184
Smooth Muscle Cells	109	1990	NA	1828
T Cells	468	867	NA	471
**T Helper Cells**	936	2491	NA	1392
T Memory Cells	269	1994	NA	798
**EGAD00001009648**	**HEALTHY**	**MGUS**	**SMM**	**NDMM**
B Cells Naive	960	6506	6041	310
Dendritic Cells	77	406	560	4
Epiblast Cells	88	498	880	37
Erythroid-like And Erythroid Precursor Cells	2539	12570	24382	376
Gamma Delta T Cells	2627	16223	28375	292

**Hematopoietic Stem Cells**	288	1395	2576	94
Luminal Epithelial Cells	209	1066	1794	43
Meningeal Cells	65	457	867	30
Neural Stem/Precursor Cells	124	789	1584	88
**Neutrophils**	1257	5471	7920	176
**NK Cells**	936	5532	8715	87
Plasma Cells	431	4692	9431	798
**Plasmacytoid Dendritic Cells**	60	190	325	10
Platelets	295	875	1242	22
Pluripotent Stem Cells	759	4334	8650	297
Podocytes	149	1179	1506	54
**Red Pulp Macrophages**	216	959	1677	29
**T Helper Cells**	961	5373	8638	30

Supplementary Table S3. Normalized cell proportions in the datasets. A. GSE124310, B. GSE163278, C. EGAD00001009648. The common cell types among the three datasets are highlighted in bold.

GSE124310	HEALTHY	MGUS	SMM	MM
B Cells Naive	0.062	0.026	0.040	0.051
Dendritic Cells	0.010	0.014	0.011	0.009
**Hematopoietic Stem Cells**	0.131	0.035	0.040	0.032
Luminal Epithelial Cells	0.075	0.012	0.018	0.026
Mesothelial Cells	0.013	0.004	0.017	0.160
**Neutrophils**	0.069	0.102	0.061	0.060
**NK Cells**	0.275	0.350	0.389	0.281
Osteoclasts	0.086	0.043	0.058	0.102
**Plasmacytoid Dendritic Cells**	0.027	0.005	0.007	0.006
**Red Pulp Macrophages**	0.051	0.085	0.062	0.064
**T Helper Cells**	0.115	0.296	0.267	0.102
T Memory Cells	0.085	0.027	0.031	0.108
**GSE163278**	**HEALTHY**	**MGUS**	**SMM**	**MM**
Adrenergic Neurons	0.093	0.136	NA	0.074
B Cells Memory	0.121	0.122	NA	0.056
Erythroid-like And Erythroid Precursor Cells	0.050	0.089	NA	0.071
Gamma Delta T Cells	0.036	0.020	NA	0.040
**Hematopoietic Stem Cells**	0.016	0.016	NA	0.039
**Neutrophils**	0.139	0.056	NA	0.100
**NK Cells**	0.268	0.170	NA	0.249
Plasma Cells	0.004	0.015	NA	0.014
**Plasmacytoid Dendritic Cells**	0.031	0.021	NA	0.026
Podocytes	0.071	0.073	NA	0.083
**Red Pulp Macrophages**	0.015	0.009	NA	0.010
Smooth Muscle Cells	0.010	0.074	NA	0.097
T Cells	0.041	0.032	NA	0.025
**T Helper Cells**	0.082	0.093	NA	0.074
T Memory Cells	0.024	0.074	NA	0.042

EGAD00001009648	HEALTHY	MGUS	SMM	MM
B Cells Naive	0.080	0.095	0.052	0.112
Dendritic Cells	0.006	0.006	0.005	0.001
Epiblast Cells	0.007	0.007	0.008	0.013
Erythroid-like And Erythroid Precursor Cells	0.211	0.183	0.212	0.135
Gamma Delta T Cells	0.218	0.237	0.246	0.105
**Hematopoietic Stem Cells**	0.024	0.020	0.022	0.034
Luminal Epithelial Cells	0.017	0.016	0.016	0.015
Meningeal Cells	0.005	0.007	0.008	0.011
Neural Stem/Precursor Cells	0.010	0.012	0.014	0.032
**Neutrophils**	0.104	0.080	0.069	0.063
**NK Cells**	0.078	0.081	0.076	0.031
Plasma Cells	0.036	0.068	0.082	0.287
**Plasmacytoid Dendritic Cells**	0.005	0.003	0.003	0.004
Platelets	0.024	0.013	0.011	0.008
Pluripotent Stem Cells	0.063	0.063	0.075	0.107
Podocytes	0.012	0.017	0.013	0.019
**Red Pulp Macrophages**	0.018	0.014	0.015	0.010
**T Helper Cells**	0.080	0.078	0.075	0.011

Supplementary Table S4. Datasets used in the study.

Three datasets were used, GSE124310 and GSE163278 datasets with only CD138-negative samples and, the EGAD00001009648 dataset containing both CD138-positive and -negative samples. Only the negative samples were retained for further analyses. *One sample not used due to low cell count; **One sample not used since the individual was in RRMM (Relapsed/Refractory Multiple Myeloma) treatment.

Accession Number	Disease Stage	Number of Samples	CD138 Status	Samples Used
**GSE124310**	Healthy donors	9	CD138-	9
	MGUS patients	5	CD138-	5
	SMM patients	11	CD138-	11
	MM patients	7	CD138-	7
**GSE163278**	Healthy donors	8	CD138-	8
	MGUS patients	14	CD138-	14
	MM patients	11	CD138-	11
**EGAD00001009648**	Healthy donors	4	1 CD138+ and 3 CD138-	3 CD138-
	MGUS patients	21	6 CD138+ and 14 CD138-	13 CD138- *
	SMM patients	32	7 CD138+ and 25 CD138-	25 CD138-
	MM patients	8	5 CD138+ and 3 CD138-	2 CD138- **

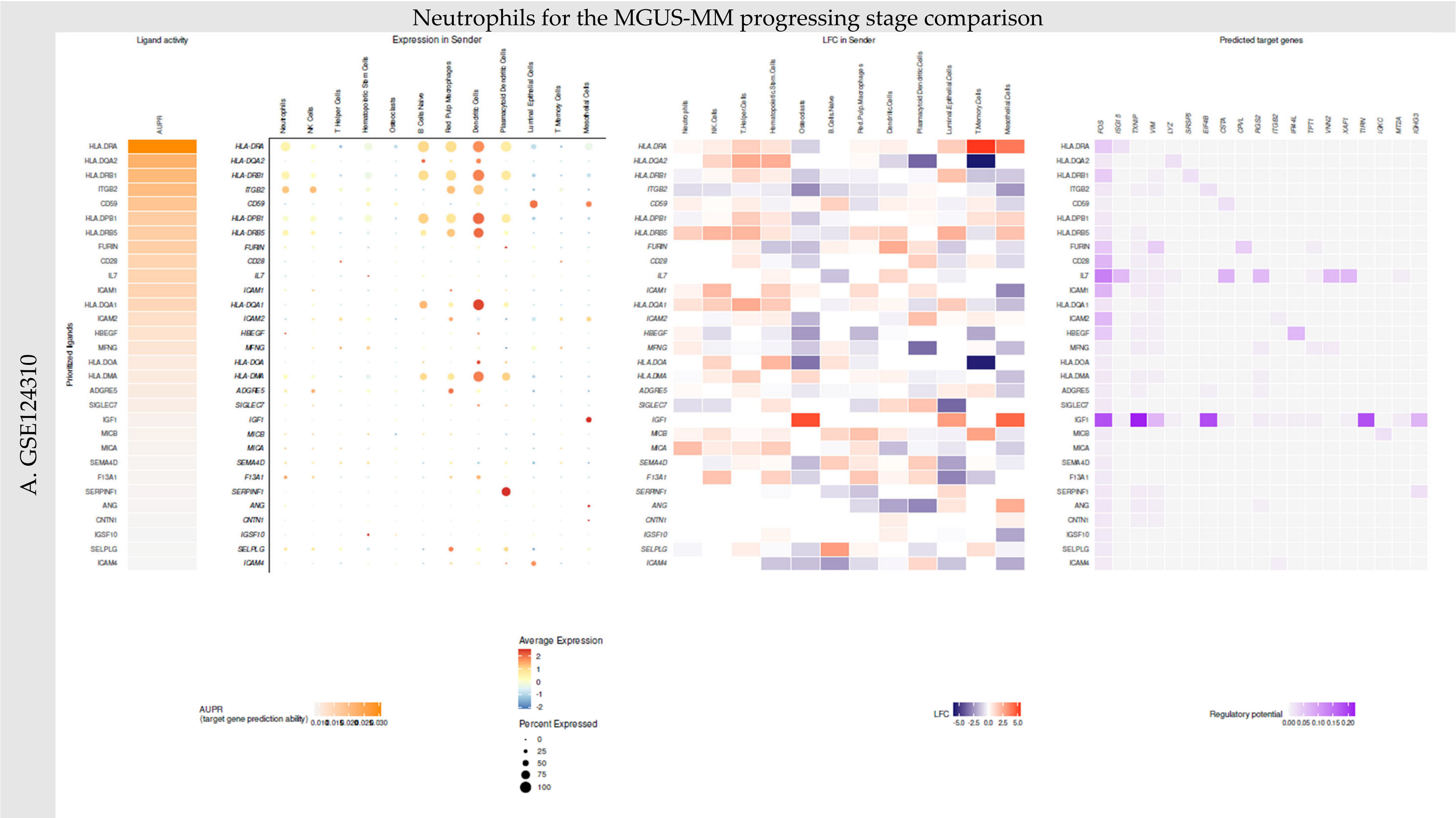

Neutrophils for the MGUS-MM progressing stage comparison
A. GSE124310
Ligand activity
Expression in Sender
LFC in Sender
Predicted target genes
Prioritized ligands
AUPR
Average Expression
Percent Expressed
LFC
Regulatory potential
AUPR (target gene prediction ability)

B. GSE163278

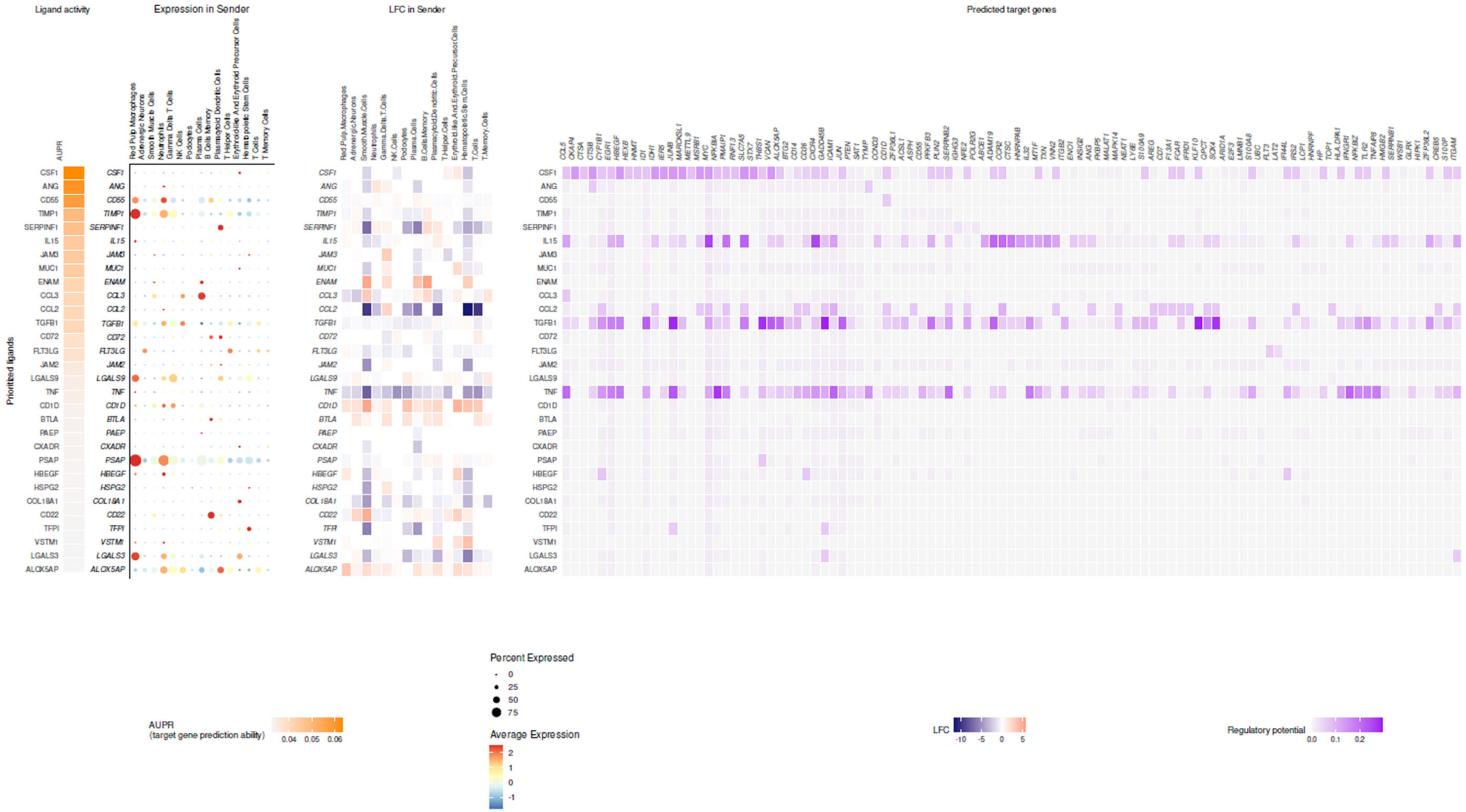
Ligand activity
Expression in Sender
LFC in Sender
Predicted target genes
AUPR
Prioritized ligands
CSF1
ANG
CD55
TIMP1
SERPINF1
IL15
JAM3
MUC1
ENAM
CCL3
CCL2
TGFB1
CD72
FLT3LG
JAM2
LGALS9
TNF
CD1D
BTLA
PAEP
CXADR
PSAP
HBEGF
HSPG2
COL18A1
CD22
TFPI
VSTM1
LGALS3
ALOX5AP
Percent Expressed
0
25
50
75
Average Expression
2
1
0
-1
AUPR
(target gene prediction ability)
0.04
0.05
0.06
LFC
-10
-5
0
5
Regulatory potential
0.0
0.1
0.2

C. EGAD00001009648

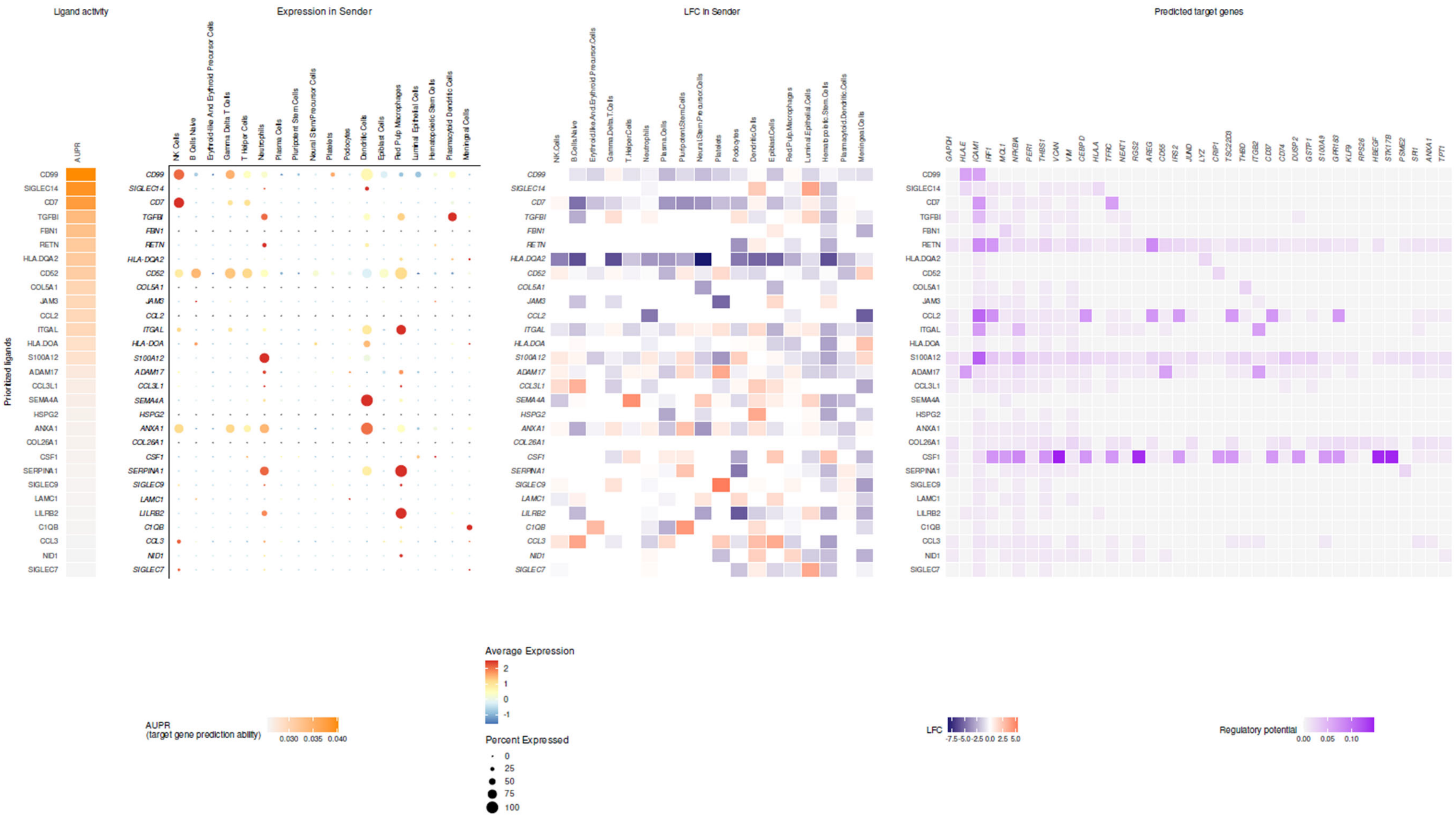

D. GSE124310

Naïve B cells for the SMM-MM progressing stage comparison

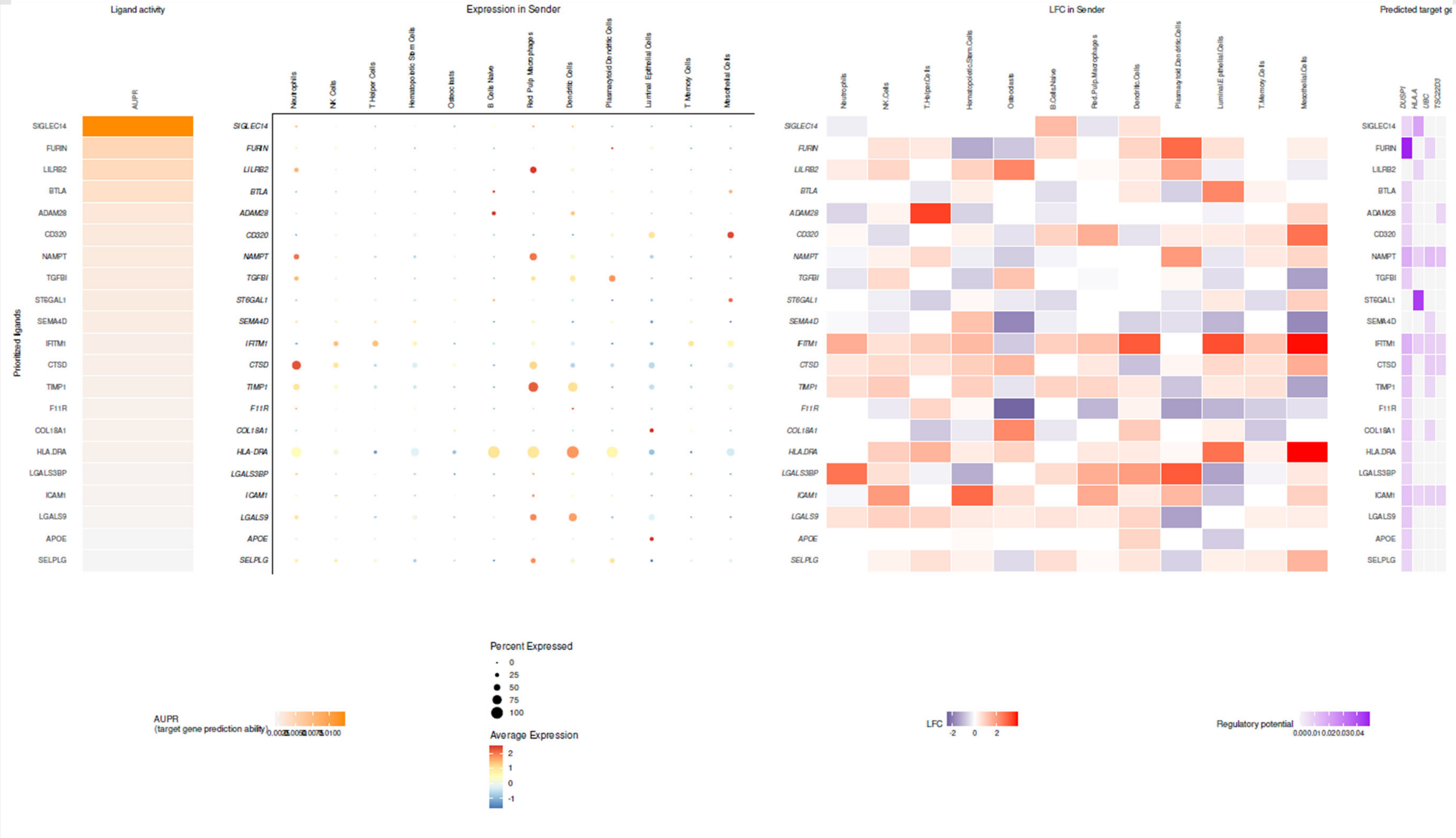

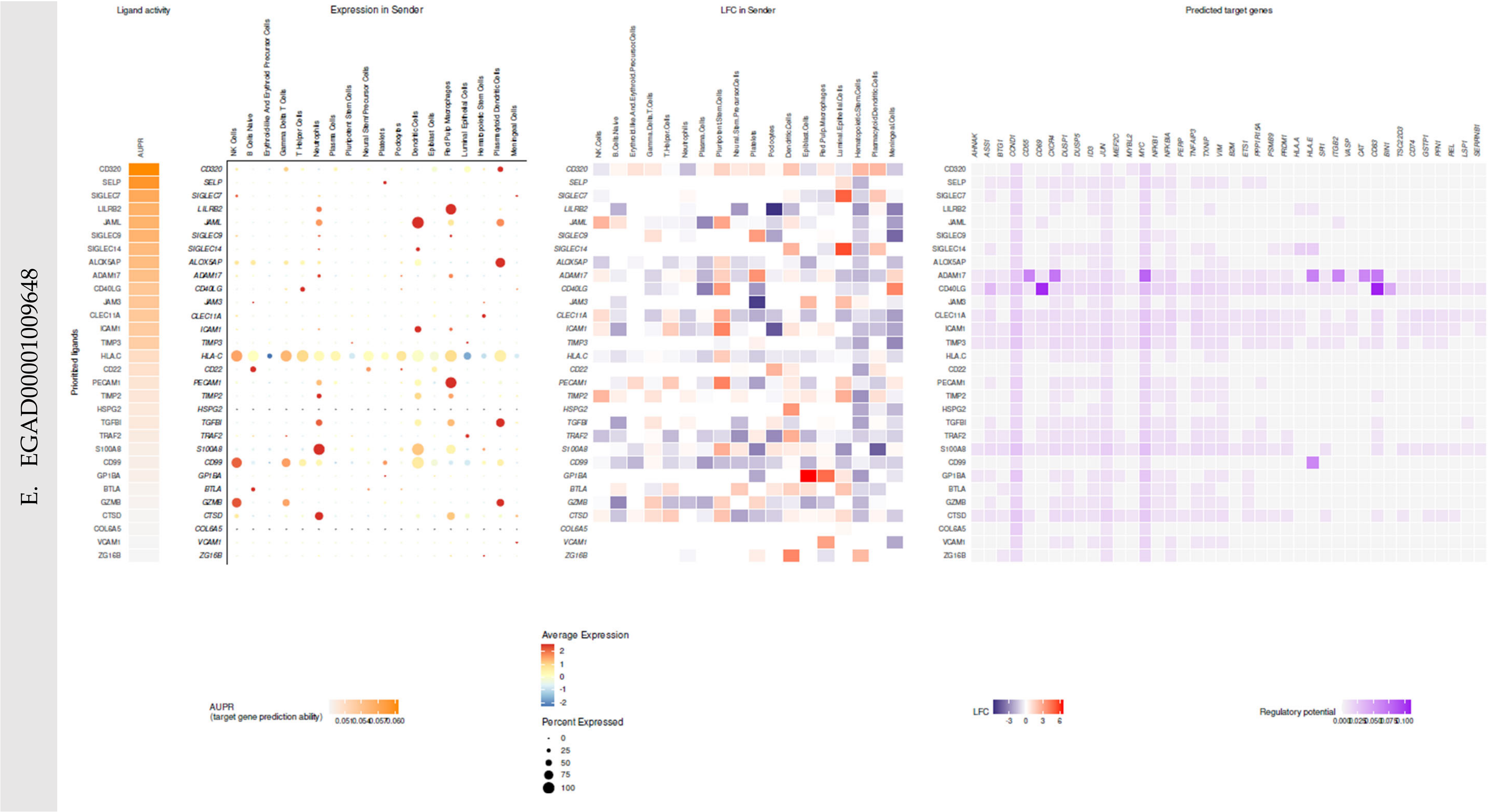

Supplementary Figure S2. Summary of NicheNet analysis for neutrophils and naïve B cells.

NicheNet results highlighting ligand activity, ligand expression, differential expression, and predicted target genes for selected receiver cell types. Results are shown for neutrophils (**A**–**C**) in MGUS-MM comparisons for datasets GSE124310, GSE163278, and EGAD00001009648, and for naïve B cells (**D**,**E**) in SMM-MM comparisons for the same

datasets. Panels display (i) top-ranked ligands based on activity scores, (ii) ligand expression levels in sender cells (dot plot indicating average expression and percentage of expressing cells), (iii) log fold change (LFC) of ligand expression between conditions, and (iv) predicted downstream target genes in receiver cells based on regulatory potential scores. LFC: log fold change and predicted downstream target genes influenced by the top-ranked ligands in the receiver cells of interest

Supplementary Reference

1. Zheng, G.X.Y.; Terry, J.M.; Belgrader, P.; Ryvkin, P.; Bent, Z.W.; Wilson, R.; Ziraldo, S.B.; Wheeler, T.D.; McDermott, G.P.; Zhu, J.; et al. Massively Parallel Digital Transcriptional Profiling of Single Cells. *Nat. Commun.* **2017**, *8*, 14049. https://doi.org/10.1038/ncomms14049.
